# Citrus Peel Extract Powders as Reducing Agents for Naturally Cured Pork Sausages: Effects on Cured Color Development

**DOI:** 10.3390/foods14081397

**Published:** 2025-04-17

**Authors:** Su Min Bae, Yeongmi Yoo, Jibin Park, Minhyeong Kim, Jong Youn Jeong

**Affiliations:** 1 Department of Food Science & Biotechnology, Kyungsung University, Busan 48434, Republic of Korea; kzm1230@naver.com (S.M.B.); pleomax159@naver.com (M.K.); 2 Brain Busan 21 Plus Project Team, Kyungsung University, Busan 48434, Republic of Korea; 3 Food & Life Science Research Institute, Kyungsung University, Busan 48434, Republic of Korea

**Keywords:** citrus peel extract, reducing agent, nitrite alternative, clean-label, cured meat color, pork sausage

## Abstract

Meat products contain synthetic additives such as sodium ascorbate and sodium erythorbate as reducing agents, raising concerns regarding their potential health implications. This study evaluated the effectiveness of grapefruit, lemon, mandarin, or orange peel extract powders (0.1% each) as natural reducing agents in pork sausages, in combination with pre-converted Chinese cabbage powder (PCCP, 0.44%) as a natural nitrite source. The physicochemical properties of the citrus extracts varied, with the lemon peel extract powder exhibiting the lowest pH (4.90) and the highest vitamin C content (874.84 mg/100 g). Sausages containing PCCP and citrus peel extract powders exhibited lower cooking loss (4.54–5.45%) than the control (5.93%), while their pH remained unaffected. Color analysis of the citrus extract-treated samples revealed no significant differences in lightness and redness and increased yellowness. The residual nitrite content was observed to be higher in the groups treated with citrus peel extract powders (53.91–54.98 ppm) compared to the groups treated with sodium ascorbate (29.88 and 34.16 ppm). However, the cured pigment content, curing efficiency, and lipid oxidation were consistent across all formulations. Our findings suggest that the use of citrus peel extract powders can replace the addition of sodium ascorbate in clean-label cured meat products without compromising color development and oxidative stability.

## 1. Introduction

The use of synthetic additives in meat products has elicited concerns regarding their potential health implications and consumer preferences. Synthetic additives, such as sodium ascorbate and sodium erythorbate, are extensively employed as cure accelerators in processed meat products, primarily functioning as reducing agents that facilitate the conversion of nitrite into nitric oxide, thereby promoting the formation of nitrosyl hemochrome for cured color stabilization [1,2]. Furthermore, these compounds exhibit antioxidant properties, which contribute to the mitigation of lipid oxidation. However, concerns regarding the long-term health effects of synthetic additives and the overall impact of nitrite usage in processed meats have driven research on natural alternatives [2,3,4].

In response to these concerns, clean-label meat products with minimal artificial additives and high quality and safety have garnered substantial interest [4,5,6]. Natural alternatives, such as vegetable powders rich in naturally occurring nitrates, polyphenols, and antioxidants, are being investigated as potential substitutes for synthetic additives [7,8,9,10]. Vegetable powders, including celery, Swiss chard, and Chinese cabbage powders, have been extensively studied as nitrate/nitrite sources in cured meat products, facilitating the formation of nitric oxide without synthetic nitrite addition [3,9,10,11,12]. Moreover, plant-based antioxidants, including polyphenolic extracts from grape seeds and green tea, have demonstrated efficacy in inhibiting lipid oxidation and extending the shelf life of meat products [13,14]. Notwithstanding these advancements, an additional curing accelerator is often necessary to enhance the reduction of nitrite to nitric oxide and improve the stability of cured color [2,12,15].

Natural cure accelerators, such as cherry and acerola powders, have been effectively utilized as reducing agents in cured meat products, serving as substitutes for synthetic sodium ascorbate and sodium erythorbate [1,12,15]. Cherry powder, derived from acerola cherry, enhances nitric oxide formation by accelerating the conversion of nitrite to nitric oxide in a manner similar to that of sodium ascorbate [12]. Furthermore, acerola powder, which contains ascorbic acid, exhibits significant antioxidant potential in nitrite-free curing systems [11,16]. In addition, citrus extracts have been explored as viable alternatives, owing to their high bioactive compound contents such as carotenoids, flavonoids, and essential oils, demonstrating antimicrobial and antioxidant properties in meat systems [16,17].

Citrus peel and its derivatives are famous for their bioactive properties, including antioxidant and antimicrobial effects [18,19,20,21,22,23]. Furthermore, citrus peel extracts are rich in polyphenols, flavonoids, and ascorbic acid, contributing to their substantial antioxidant potential [19,20,24]. Notably, flavonoids, such as hesperidin and naringin, exhibit metal-chelating and radical-scavenging activities that may enhance nitric oxide formation from nitrite [14,25,26,27]. Previous studies have suggested that citrus extracts or co-products, particularly those from lemons and oranges, may function as reducing agents by enhancing the conversion of nitrite to nitric oxide [17,28]. However, the precise mechanisms by which citrus polyphenols contribute to this reaction remain unclear and require further investigation. Such findings suggest that citrus-derived extracts could serve as natural cure accelerators in the development of clean-label meat products, owing to their rich ascorbic acid and polyphenol contents. In addition to their functional properties, citrus peels are by-products of the juice and food processing industries, rendering them economically and environmentally sustainable [21,24]. Their use in meat products not only adds value to food waste but also aligns with sustainability objectives by repurposing agricultural by-products into functional ingredients.

Although previous studies have suggested the potential of citrus peel extracts for nitrite reduction and color stabilization, the evaluation of different citrus species (grapefruit, lemon, mandarin, and orange) combined with vegetable-derived nitrite sources remains understudied. Specifically, Jeong et al. [10] demonstrated that Chinese cabbage powder can function as a natural nitrate/nitrite source; however, its interaction with citrus peel extracts in alternative curing systems has not been thoroughly investigated. The combination of citrus-derived bioactive compounds with vegetable-based nitrite sources should be further studied, to explore whether they synergistically enhance nitrite reduction. Owing to their high ascorbic acid and polyphenol contents, citrus peel extracts show great promise for enhancing nitrite reduction and improve cured color stability, thereby rendering them viable replacements for synthetic ascorbates in naturally cured meat products.

Therefore, in this study, we aimed to investigate the potential of citrus peel extract powders as natural cure accelerators in conjunction with pre-converted Chinese cabbage powder (PCCP) to develop clean-label pork sausages.

## 2. Materials and Methods

### 2.1. Citrus Peel Extract Powder Preparation

Citrus peel extract powders were prepared by washing the peels of fresh grapefruit, lemons, mandarins, and oranges, followed by drying at 40 °C for 12 h using a food dryer (EN-FO-392S, Enex Science, Goyang, Republic of Korea). The dried peels were ground into a fine powder (moisture content < 5%) and mixed with 50% ethanol (1:50, *w*/*v*) under continuous stirring (100 rpm) at 80 °C for 30 min. The extracts were rapidly cooled using chilled water and centrifuged at 3400× *g* for 10 min (Combi R515, Hanil Scientific, Gimpo, Republic of Korea). The supernatants were filtered through filter paper (1001-110, Cytiva, Little Chalfont, UK) and were concentrated under vacuum using a rotary evaporator. The concentrated extracts were freeze-dried at −40 °C under 5 Pa for 48 h and ground into a fine powder. The resulting grapefruit peel extract powder (GPEP), lemon peel extract powder (LPEP), mandarin peel extract powder (MPEP), and orange peel extract powder (OPEP), were vacuum-sealed in polyethylene bags and stored in aluminum-sealed packaging at −24 °C until use.

### 2.2. Pre-Converted Chinese Cabbage Powder Preparation

Chinese cabbage was sourced from local markets (Busan, Republic of Korea), washed, and cut into small pieces. The juice was extracted following a cold-press extraction method using a juice extractor (Juice Extractor 68, Santos, Vaulx-en-Velin, France), followed by centrifugation at 3400× *g* for 10 min, and filtration of the supernatant through a 75 µm sieve. The natural nitrate contained in the Chinese cabbage extract was converted to nitrite using *Staphylococcus hominis* subsp. isolated from kimchi as the starter culture [29]. Thus, the Chinese cabbage extract was mixed with 0.1% starter culture (10^9^ CFU/g), 1% glucose, and 0.06% calcium carbonate, and incubated at 37 °C for 9 h upon shaking at 100 rpm. The fermented extract was heated at 65 °C for 30 min, cooled, and subsequently freeze-dried [30]. The freeze-dried extract was then ground into a fine powder. PCCP was analyzed for nitrate and nitrite ions, followed by vacuum-sealing and storage in aluminum-sealed packaging at −24 °C until use.

### 2.3. Preparation of Raw Materials and Ground Pork Sausages

Fresh pork ham (*M. biceps femoris*, *M. semitendinosus*, and *M. semimembranosus*) and back fat were obtained from a local processor (Pukyung Pig Farmers Livestock, Gimhae, Republic of Korea) within 24−48 h postmortem. Visible connective tissues were removed, and the meat was cut into cubes of approximately 5 × 5 × 5 cm. Pork meat and back fat were subsequently ground using 8 mm and 3 mm plates (TC-22 Elegant plus, Tre Spade, Torino, Italy).

The basic formulation for the sausage preparation included 70% pork ham, 15% back fat, 15% water/ice, 2% NaCl, and 1% sugar. Six experimental groups were prepared: control, 0.01% sodium nitrite and 0.01% sodium ascorbate; PSA, 0.44% PCCP and 0.01% sodium ascorbate; GPEP, 0.44% PCCP and 0.1% GPEP; LPEP, 0.44% PCCP and 0.1% LPEP; MPEP, 0.44% PCCP and 0.1% MPEP; and OPEP, 0.44% PCCP and 0.1% OPEP. The addition of 0.44% PCCP was based on the sodium nitrite content (22,970 ppm) of PCCP prepared for this investigation. This concentration was selected to correspond to the equivalent amount of 0.01% synthetic sodium nitrite. A 0.1% incorporation of citrus peel extract powders was applied, in accordance with a prior study [11] that employed fruit-derived powders as natural curing accelerators in meat products.

The ground pork was mixed with NaCl and a nitrate source (either sodium nitrite or pre-converted Chinese cabbage powder) in a mixer (5K5SS, Whirlpool, St. Joseph, MI, USA) at 120 rpm for 4 min. Back fat, sugar, and sodium ascorbate or the corresponding citrus peel extract powder (depending on treatment group) was then added, followed by another 4 min of mixing. The mixture was stuffed into 24 mm cellulose casings (Viskase^®^ Companies, Lombard, IL, USA) using a stuffer, followed by overnight curing at 3 °C. The sausages were cooked in a water bath at 90 °C until a core temperature of 75 °C, followed by rapid cooling in ice-cold water for 20 min, and storage at 3 °C until further analysis. Processing of the cured sausage was independently replicated three times on separate days.

### 2.4. Citrus Peel Extract Powder and Pre-Converted Chinese Cabbage Powder Analysis

The pH of the citrus peel extract powder was measured by mixing the powder with distilled water in a 1:9 weight ratio. The mixture was homogenized using a vortex mixer for 1 min, and the pH was determined using a pH meter (Accumet^®^ AB150, Thermo Fisher Scientific, Singapore).

The total phenolic content was analyzed using the method of Singleton et al. [31] with slight modifications. The citrus peel extract powder was diluted 1:1000 in distilled water, and 0.4 mL of the sample was mixed with 2 mL of 10% Folin-Ciocalteu reagent. After reacting for 5 min, 1.6 mL of a 7.5% sodium carbonate solution was added. The mixture was incubated for 120 min at room temperature and the absorbance was measured at 765 nm using a spectrophotometer (UV-1800, Shimadzu, Kyoto, Japan). Gallic acid (G7384, Sigma-Aldrich, St. Louis, MO, USA) was used as a standard, and the results were expressed as mg gallic acid equivalents (GAE) per gram of sample.

The vitamin C content was quantified using the method of Nielsen [32] with slight modifications. The citrus peel extract powder was mixed with distilled water at a 1:100 weight ratio, and 2 mL of the mixture was combined with 5 mL of the metaphosphoric acid-acetic acid solution. The mixture was titrated with an indophenol solution until a stable pink color persisted for at least 5 s. L-Ascorbic acid (#35268, Acros Organics, Geel, Belgium) was used as the reference standard and the results are expressed as mg ascorbic acid (AA) per 100 g of sample.

The nitrate and nitrite contents of the pre-converted Chinese cabbage powder were determined using the zinc reduction method described by Merino [33]. The samples were homogenized in distilled water and filtered. The nitrate content was determined using zinc to reduce nitrate to nitrite, which was then quantified by reacting with sulfanilamide and N-(1-naphthyl) ethylenediamine dihydrochloride. The nitrite content was directly analyzed using the Griess reaction and the absorbance was measured at 540 nm. The results are expressed as ppm (mg/kg) of nitrate and nitrite ions.

### 2.5. Determination of Cooking Loss and pH in Cured Pork Sausages

To determine cooking loss, the weight of the meat mixtures in the cellulose casings was recorded prior to cooking. After cooking and cooling, as described in Section 2.3, the sausages were re-weighed, and any excess moisture was removed using paper towels. The cooking loss was determined as a percentage by comparing the pre- and post-cooking weights. This measurement involved five sausages per treatment for each replicate. pH was assessed using a calibrated pH meter after combining the cooked sausages (5 g) with 25 mL of distilled water and homogenizing. Two sausages from each treatment in every replication were analyzed, with two pH readings taken per sample.

### 2.6. Instrumental Color Measurement in Cured Pork Sausages

A chromameter (CR-400, Konica Minolta Sensing, Osaka, Japan) was used with an 8 mm aperture, illuminant D_65_, and a 2° observer angle to assess color using the CIE L*a*b* system. The device was calibrated using a white tile prior to measurements. Instrumental color was measured after all samples were kept at room temperature for 1 h. To obtain the CIE color readings, each sausage sample was sliced lengthwise, and three measurements were taken randomly on both sides of the cut surface immediately after cutting to prevent color fading [34]. Six readings were recorded for each treatment per sample during each replication.

### 2.7. Residual Nitrite Analysis in Cured Pork Sausages

The residual nitrite was measured using the AOAC method 973.31 [35]. Homogenized samples were extracted in hot-distilled water, followed by filtration and reaction with sulfanilamide and N-(1-naphthyl) ethylenediamine dihydrochloride. The absorbance was measured at 540 nm using a spectrophotometer. The residual nitrite concentrations were calculated using a standard curve created with a sodium nitrite (S2252, Sigma-Aldrich, St. Louis, MO, USA) solution and are expressed in parts per million (ppm). Two sausage samples from each treatment in every replicate were analyzed for their residual nitrite content.

### 2.8. Cured Meat Pigment, Total Pigment, Curing Efficiency, and Cured Color Intensity in Cured Pork Sausages

Analysis of the cured meat pigment and total pigment contents was performed following the methodology outlined by Hornsey [36]. In brief, 10 g of cured sausages was blended with 80% acetone (for cured meat pigment) or acidified acetone (for total pigment) for 15 min or 1 h, respectively, followed by filtration. The absorbance of the filtered sample solution was measured at 540 nm (A540) for the cured meat pigment and 640 nm (A640) for the total pigment. The cured meat pigment was determined by multiplying A540 by 290, while the total pigment was calculated by multiplying A640 by 680. The results are expressed in ppm. For each treatment and replication, two sausage samples were used to assess the cured meat pigment and total pigment. The curing efficiency was evaluated by calculating the percentage of the cured meat pigment in relation to the total pigment content [34]. The evaluation of the cured color intensity involved determining the reflectance ratio (%R650/%R570) by examining the reflectance of the internal surface of the sample immediately after cutting, using a spectrophotometer (UV-2600i, Shimadzu, Kyoto, Japan) equipped with a multipurpose sample compartment (MPC-2600A, Shimadzu, Kyoto, Japan). The reflectance of each sample was scanned over a wavelength range of 400–700 nm, employing 1 nm increments. Subsequently, the ratio was calculated based on the acquired reflectance data.

### 2.9. Lipid Oxidation in Cured Pork Sausages

The thiobarbituric acid reactive substances (TBARS) were measured using the method described by Tarladgis et al. [37]. Following distillation, the samples were combined with 0.02 M 2-thiobarbituric acid and heated in boiling water for 35 min. The absorbance was measured at 538 nm and lipid oxidation was reported as mg malondialdehyde (MDA) per kg of sample. Two sausages were used for the TBARS analysis for each treatment across all replicates.

### 2.10. Statistical Analysis

Each experiment was conducted in triplicate on separate days. As described in Section 2.3, the experimental design of this study employed a randomized block design with six groups (control, PSA, GPEP, LPEP, MPEP, and OPEP). Statistical analysis of the data was performed using the Proc GLM (general linear model) procedure of SAS software (version 9.4, SAS Institute, Cary, NC, USA). Significant differences (*p* < 0.05) were further evaluated using Duncan’s multiple range test.

## 3. Results and Discussion

### 3.1. Physicochemical Properties of Citrus Peel Extract Powders and Nitrite Content of Pre-Converted Cabbage Powder

#### 3.1.1. pH, Total Phenolic Content, and Vitamin C Content of Citrus Peel Extract Powders

The pH, total phenolic content, and vitamin C content of the citrus peel extract powders are presented in Table 1. LPEP exhibited the lowest (*p* < 0.05) pH value (pH 4.90), whereas MPEP had the highest (*p* < 0.05, pH 5.38), indicating variations in the acidity levels due to differences in the organic acid content. These pH values differed from those reported by Koutoulis et al. [38], where mandarin and lemon peel extracts exhibited higher pH levels. This discrepancy may be attributed to differences in the extraction methods, sample preparation, or measurement conditions. Nevertheless, previous studies indicate that mandarin peel extracts typically exhibit a lower total titratable acidity than lemon and orange peels, which may explain the relatively higher pH observed in this study [38]. The predominant acids contributing to these differences include citric, malic, and oxalic acids, exhibiting various concentrations among citrus species [22].

The highest total phenolic content (TPC) was observed in GPEP (107.63 mg GAE/g, *p* < 0.05) and the lowest was observed in MPEP (53.46 mg GAE/g, *p* < 0.05) and OPEP (53.29 mg GAE/g, *p* < 0.05), confirming the presence of bioactive compounds that contribute to their antioxidant properties. Previous studies have also reported variations in TPC among citrus peel extracts depending on the extraction method and citrus species [19]. Compared with water-based extraction solvents, ethanol has been shown to improve polyphenol solubility; specifically, extraction with 50% ethanol has effectively preserved polyphenols [19]. Furthermore, citrus albedo tissue is particularly rich in flavonoids and polyphenols, further enhancing the antioxidant potential of citrus peel extracts [25].

In this study, the vitamin C content of citrus peel extracts was also analyzed, with LPEP showing the highest (*p* < 0.05) concentration (874.84 mg AA/100 g), followed by MPEP (556.43 mg AA/100 g). GPEP (441.36 mg AA/100 g) and OPEP (455.55 mg AA/100 g) exhibited the lowest concentration (*p* < 0.05). A previous study suggested that citrus peels are a valuable source of natural antioxidants, including vitamin C [38]. However, the stability of vitamin C is highly dependent on the processing method used. Furthermore, high temperatures and prolonged storage lead to a significant degradation of ascorbic acid [22]. In this study, vacuum freeze-drying was used for powder preparation because it has been shown to effectively preserve antioxidants, including vitamin C.

#### 3.1.2. Nitrite Content of Pre-Converted Chinese Cabbage Powder

Fermentation using nitrate-reducing bacteria has been shown to facilitate nitrite formation from nitrate-rich vegetables, thereby enhancing its efficacy as an alternative curing agent [12,29,39]. Certain strains, such as *Staphylococcus hominis*, isolated from fermented foods, have demonstrated significant nitrate reduction capabilities, contributing to natural nitrite formation [29]. In this study, nitrate contained in the Chinese cabbage extract exhibited significant nitrite conversion after fermentation in the presence of a starter culture. Upon powdering, PCCP contained nitrate and nitrite ion concentrations of 17,030 and 15,298 ppm, respectively, which corresponded to 23,329 and 22,970 ppm of sodium nitrate and sodium nitrite, respectively. Sebranek et al. [40] reported that commercially available pre-converted vegetable products, such as celery powder, generally contain nitrite levels ranging from 15,000 to 20,000 ppm. Such a finding suggests that the developed PCCP is a suitable nitrite alternative for meat products. Furthermore, recent studies have indicated that pre-fermented vegetable powders, including those derived from Chinese cabbage, can provide a consistent and controlled source of nitrite, ensuring predictable curing effects in meat products [10,30].

### 3.2. Effects of Citrus Peel Extract Powders as Reducing Agents on the Physicochemical Characteristics of Ground Pork Sausages Cured with Pre-Converted Chinese Cabbage Powder

#### 3.2.1. Cooking Loss and pH

Compared with the control (using synthetic sodium nitrite and ascorbate), the cooking loss was significantly lower (*p* < 0.05) in all the treatments using PCCP and citrus peel extract powders (Table 2). This improvement in cooking yield may be attributed to the high water retention capacity of the dietary fibers from Chinese cabbage and citrus peel, which help retain moisture and fat during cooking, thereby reducing drip loss [41,42,43,44]. Among the sausages treated with PCCP, the PSA, GPEP, and LPEP treatments exhibited no significant differences (*p* > 0.05) in cooking loss; however, these treatments exhibited lower (*p* < 0.05) cooking loss than the OPEP treatment.

The pH values of all the sausage samples did not show significant differences (*p* > 0.05, Table 2), indicating that PCCP and the type of citrus peel extract powders did not influence the overall pH of the product. Although the added citrus peel extract powders exhibited mild acidity (pH 4.90–5.38, Table 1), the inherent buffering capacity of meat facilitated pH stabilization against external acidic or alkaline influences [45,46]. Moreover, previous studies revealed that meat products formulated with natural curing agents, such as celery and cherry powders, can maintain pH levels comparable to those of conventional nitrite-cured sausages [1,12].

#### 3.2.2. Instrumental Color

Instrumental color analysis revealed significant differences in the CIE L*, a*, and b* values among the treatment groups (Table 2). Compared with the control, the PSA, GPEP, and LPEP treatments exhibited similar (*p* > 0.05) CIE L* values. However, they did not differ (*p* > 0.05) significantly from MPEP and OPEP; such a finding aligns with previous reports on naturally cured meat products. According to Choi et al. [11], the CIE L* values of indirectly cured meat products with natural ingredients, such as white kimchi powder and acerola juice powder, did not differ significantly from those in sodium nitrite-treated controls. Furthermore, Posthuma et al. [15] reported that nitrite sources (sodium nitrite or celery juice powder) and reducing compounds (sodium erythorbate or ascorbic acid from cherry powder) did not significantly affect the L* values in cured beef sausages. Nonetheless, the CIE L* values of the MPEP and OPEP groups were significantly lower (*p* < 0.05) than those of the control group, despite their small numerical difference.

The CIE a* values indicate the redness of the sausages and did not differ significantly (*p* > 0.05) among all treatments (Table 2), including the control, PSA, and citrus peel extract powder groups (GPEP, LPEP, MPEP, and OPEP). This suggests that the incorporation of citrus peel extract powders as reducing agents along with PCCP for nitrite replacement, contributed to the formation of the pinkish-red cured color typically associated with nitrosyl hemochrome. Such a finding aligns with the cured meat pigment results obtained in this study (Table 3). Similarly, Choi et al. [11] and Posthuma et al. [15] reported that naturally derived curing agents maintained stable CIE a* values comparable to those of traditional curing methods. Previous studies have shown that plant-based ingredients, particularly those rich in polyphenols and antioxidants, can maintain the red color of cured meat products [14,47,48]. Ahmad et al. [47] and Papuc et al. [14] further highlighted that fruit-based antioxidants, including citrus extracts, stabilize meat pigments by preventing oxidative degradation. Therefore, the antioxidant properties and reducing capacity of the citrus peel extract powders possibly contribute to the color stability, supporting their potential use as natural curing accelerators for clean-label meat products.

The comparison between the control and PSA treatment, both containing sodium ascorbate as a reducing agent, revealed no statistically significant differences (*p* > 0.05) in the CIE b* values (Table 2), regardless of the nitrite source used (sodium nitrite versus natural nitrite derived from PCCP). In a related study, Jeong et al. [9] examined cured products using Chinese cabbage powder and radish powder as alternative nitrate sources, in conjunction with nitrate-reducing bacteria. Their findings indicated that while the radish powder treatment yielded CIE b* values comparable to those of sodium nitrite-added sausages, the Chinese cabbage powder treatment resulted in increased yellowness levels. However, the CIE b* values were significantly higher (*p* < 0.05) in the samples containing citrus peel extract powders compared with the control and PSA treatments. This observation can be attributed to the presence of citrus peel-derived pigments, such as carotenoids and flavonoids [21,24,26], which may impart a yellowish color to the final product. Choi et al. [49] observed that the addition of red beet extract increased the CIE b* values in emulsified sausages, supporting the hypothesis that plant-derived pigments contribute to the yellowness of cured meat products. Among the citrus peel extract powder-treated sausages, the OPEP treatment exhibited CIE b* values comparable to those of the GPEP and LPEP treatments (*p* > 0.05); however, it exhibited significantly lower (*p* < 0.05) CIE b* values compared with the MPEP treatment. The reduced CIE b* values in the OPEP treatment relative to the MPEP treatment indicate that the orange peel extract contains less orange-yellow pigments than the mandarin peel extract, which is characterized by its higher β-cryptoxanthin content [21,24].

#### 3.2.3. Residual Nitrite Content, Cured Meat Pigment, Total Pigment, Curing Efficiency, and Cured Color Intensity

Table 3 presents the residual nitrite content, cured meat pigment (nitrosyl hemochrome), total pigment, curing efficiency, and cured color intensity in the cured pork sausages. Residual nitrite plays a crucial role in the color development and antimicrobial properties of cured meat products. In clean-label processed meats, maintaining an appropriate level of residual nitrite is essential for ensuring both product safety and quality [3,4]. In this study, cured pork sausages containing citrus peel extract powders (GPEP, LPEP, MPEP, and OPEP) did not exhibit significant differences (*p* > 0.05) in their residual nitrite content (Table 3). However, they exhibited significantly higher (*p* < 0.05) residual nitrite levels than both the traditionally cured control and the PSA treatment. This finding suggests that citrus peel extracts have a limited nitrite-reducing capacity compared with sodium ascorbate. Sodium ascorbate is a highly efficient and pure reducing agent known to accelerate the conversion of nitrite to nitric oxide, which is a crucial step in cured meat pigment formation [4,48]. The lower efficiency of citrus peel extracts for nitrite reduction may be attributed to their complex antioxidant composition, which includes polyphenols and flavonoids that may not function as direct reducing agents. Unlike sodium ascorbate, which acts as a strong and direct nitrite reducer, citrus-derived antioxidants may interact differently with nitrite, potentially slowing its conversion to nitric oxide [28]. Interestingly, previous studies have reported that naturally cured meat products often exhibit lower residual nitrite levels than traditionally cured counterparts, necessitating supplementary strategies to maintain their color stability and microbial safety during storage [4,50,51]. However, the citrus peel extracts may influence nitrite retention due to their specific antioxidant composition, which differs from that of pure sodium ascorbate as a reducing agent. From a practical perspective, the higher residual nitrite in citrus-treated sausages may serve as a reservoir for the regeneration of the cured meat color during storage [52], potentially enhancing the color stability over time. This suggests that citrus peel extract powders not only act as natural curing accelerators but also help maintain the visual appeal of clean-label meat products.

Cured pigment formation (nitrosyl hemochrome) is a key indicator of the color stability in cured meat products [34]. In this study, despite the higher residual nitrite in sausages containing PCCP and citrus peel extract powders, the cured meat pigment was not significantly different (*p* > 0.05) among all sausage formulations, including the control, PSA, and citrus peel extract powder-treated groups (GPEP, LPEP, MPEP, and OPEP, Table 3). This finding implies that citrus peel extract powders exhibit a reducing capability and promote nitrosyl hemochrome formation, indicating that PCCP and the citrus peel extracts can effectively replace synthetic nitrite and ascorbate. Similarly, previous studies showed that the substitution of sodium nitrite and sodium erythorbate with celery juice and cherry powders afforded comparable cured meat pigment in meat products [12]. Furthermore, the use of white kimchi and acerola juice powders as replacements for sodium nitrite and sodium ascorbate, respectively, has been reported to develop a cured color similar to conventionally cured products [11]. These findings align with those of our study, indicating that naturally derived compounds can afford cured meat pigment formation without the use of synthetic additives. In addition, citrus co-products have been explored as potential ingredients to modulate the residual nitrite levels in meat products, potentially influencing the availability of nitric oxide for cured meat pigment formation [28]. However, the efficiency of phenolic compounds in promoting nitrite reduction depends on their structural characteristics and pH conditions, which influence their role as reducing agents [2]. Therefore, our findings indicate that citrus peel extracts combined with PCCP maintained similar nitrosyl hemochrome levels despite the absence of sodium ascorbate, being highly promising as natural curing agents in clean-label meat products without compromising the stability of the cured meat pigment.

The total pigment content of the cured sausages was not significantly affected (*p* > 0.05) by the type of citrus peel extract powders (GPEP, LPEP, MPEP, and OPEP, Table 3). The GPEP and OPEP treatments exhibited no significant differences (*p* > 0.05) in the total pigment content compared with the PSA treatment, however, the contents were lower (*p* < 0.05) than that of the control. The numerical values for these treatments were also minimal compared with those of the control. Such findings indicate that replacing sodium ascorbate with citrus peel extract powders combined with PCCP did not negatively affect the total pigment, suggesting that citrus peel extracts can serve as effective natural curing agents. The observed stability of the total pigment content is consistent with previous studies reporting that natural curing agents, such as pre-converted vegetable powders, do not drastically alter the overall pigment composition [9,39]. Similar trends have been reported for celery juice and acerola juice powders, where the total pigment content remained similar to that of conventionally cured products [11]. Additionally, the relationship between the total pigment content and nitrosyl hemochrome formation has been well documented [9,34,53]. Increased nitrosoheme pigment formation leads to a higher total pigment content, which supports the role of nitrite-derived curing reactions in total pigment stabilization [9,53]. Furthermore, alternative curing methods utilizing plant-derived nitrites have been shown to contribute to the stabilization of cured meat pigment by preventing oxidative degradation [51]. Consequently, these findings demonstrate that the combination of citrus peel extracts with PCCP effectively preserves the total pigment content in cured meat products, similar to the effects observed with sodium ascorbate.

The curing efficiency is defined as the proportion of nitrosyl hemochrome relative to the total pigment and is a crucial indicator of the effectiveness of the curing agents in meat products. In this study, all treatments containing citrus peel extract powders exhibited curing efficiencies (*p* > 0.05) comparable to those containing sodium ascorbate (control and PSA), with values ranging from 73.75% to 79.53% (Table 3). These results indicate that the inclusion of citrus peel extract powders combined with PCCP effectively promoted the development of nitrosyl hemochrome, regardless of the extract type. The stability of the curing efficiency observed in citrus peel-treated sausages aligns with prior studies on naturally derived curing agents. Choi et al. [11] reported that sausages cured with white kimchi and acerola juice powders exhibited curing efficiencies between 75.26% and 79.34%, which are comparable to our findings. Similarly, Bae et al. [17] reported that lemon extract powder in naturally cured sausages maintained a curing efficiency of above 77%, reinforcing the role of citrus-derived compounds in stabilizing the cured meat color. Serdaroğlu et al. [39] showed that the use of plant-derived extracts, such as arugula and barberry extracts, in cooked fermented sausages enhanced the nitroso-pigment formation and curing efficiency, further supporting the potential role of citrus polyphenols in nitrosyl hemochrome stabilization. Thus, our findings support the potential of citrus peel extract powders as viable replacements for synthetic ascorbate in clean-label meat products. Their ability to maintain the curing efficiency at levels comparable to those of sodium ascorbate suggests their functional efficacy in preserving the cured meat color and nitrosyl hemochrome stability, providing a feasible solution for natural curing strategies in the meat industry.

**Figure 1:**
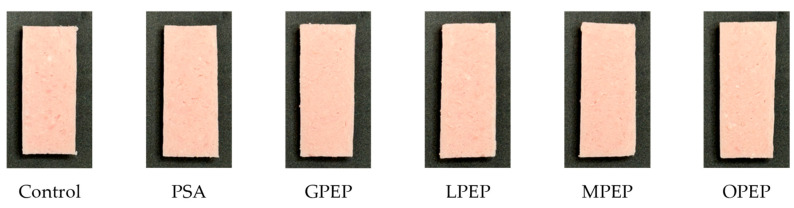
Cross-sectional comparison of sausages containing sodium nitrite and sodium ascorbate or pre-converted Chinese cabbage powder and various citrus peel extract powders: Control, 0.01% NaNO_2_ and 0.01% sodium ascorbate; PSA, 0.44% pre-converted Chinese cabbage powder and 0.01% sodium ascorbate; GPEP, 0.44% pre-converted Chinese cabbage powder and 0.1% grapefruit peel extract powder; LPEP, 0.44% pre-converted Chinese cabbage powder and 0.1% lemon peel extract powder; MPEP, 0.44% pre-converted Chinese cabbage powder and 0.1% mandarin peel extract powder; OPEP, 0.44% pre-converted Chinese cabbage powder and 0.1% orange peel extract powder.

**Figure 2:**
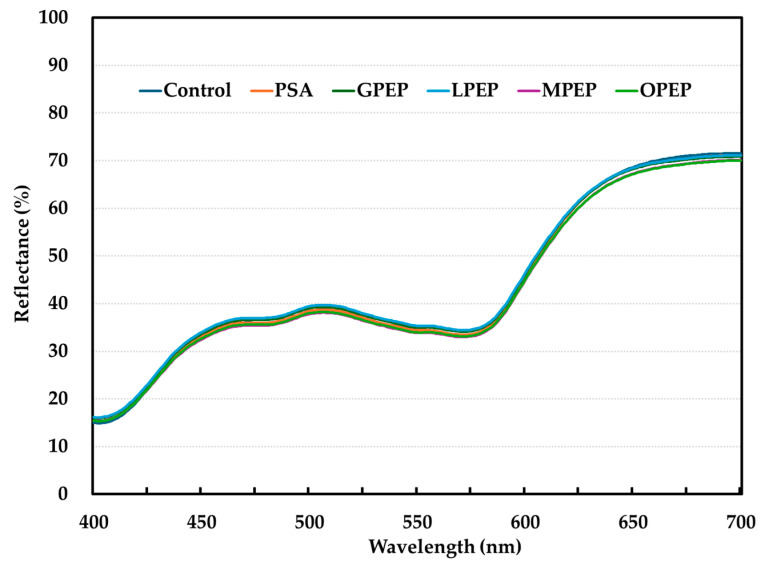
Reflectance spectra of pork sausages containing sodium nitrite and sodium ascorbate or pre-converted Chinese cabbage powder and various citrus peel extract powders: Control, 0.01% NaNO_2_ and 0.01% sodium ascorbate; PSA, 0.44% pre-converted Chinese cabbage powder and 0.01% sodium ascorbate; GPEP, 0.44% pre-converted Chinese cabbage powder and 0.1% grapefruit peel extract powder; LPEP, 0.44% pre-converted Chinese cabbage powder and 0.1% lemon peel extract powder; MPEP, 0.44% pre-converted Chinese cabbage powder and 0.1% mandarin peel extract powder; OPEP, 0.44% pre-converted Chinese cabbage powder and 0.1% orange peel extract powder.

The %R650/%R570 ratio is a key parameter for the cured color intensity, with higher values indicating a more intense cured color. This ratio is primarily influenced by the formation of nitrosyl hemochrome, which develops through the reaction of nitric oxide with myoglobin, thereby stabilizing the characteristic cured meat color [34]. In this study, the cured color intensity exhibited no significant differences (*p* > 0.05), as indicated by the %R650/%R570 ratios of the control (2.07), PSA (2.05), and citrus peel extract powder treatments (GPEP, LPEP, MPEP, and OPEP; ranging from 1.99 to 2.04, Table 3). These results indicate that the incorporation of citrus peel extract powders as a substitute for sodium ascorbate, in combination with PCCP, effectively maintained the development of cured color in pork sausages. The antioxidant properties of citrus peel polyphenols may help stabilize nitric oxide, facilitating its interaction with myoglobin to form nitrosyl hemochrome and thus preserving the cured color intensity of pork sausages. This finding was corroborated by the chemical analysis of the cured meat pigments, supporting the viability of these compounds as alternative natural curing agents. In this study, the reflectance ratio of %R650/%R570 ranged from 1.99 to 2.04% for all sausages containing PCCP and citrus peel extract powders. According to King et al. [34], a surface reflectance ratio of approximately 1.7 to 2.0 is considered indicative of a noticeable cured color. Consequently, cured pork sausages containing citrus peel extracts as reducing agents combined with PCCP exhibited an excellent cured color without requiring sodium ascorbate. Furthermore, as shown in Figure 1, visual assessment of the cured sausages confirmed the instrumental findings, showing no discernible differences in the cured color intensity across all samples. In addition, the reflectance spectra of all the tested samples exhibited a consistent pattern (Figure 2). This finding indicates that the curing process proceeds uniformly, regardless of the type of citrus peel extract powder as well as the nitrite source (sodium nitrite vs. PCCP). Furthermore, the combination of citrus peel extracts with PCCP effectively supports the development of cured color and stability in clean-label meat products, reinforcing their potential as natural curing agents without compromising the visual quality.

#### 3.2.4. Lipid Oxidation

**Figure 3:**
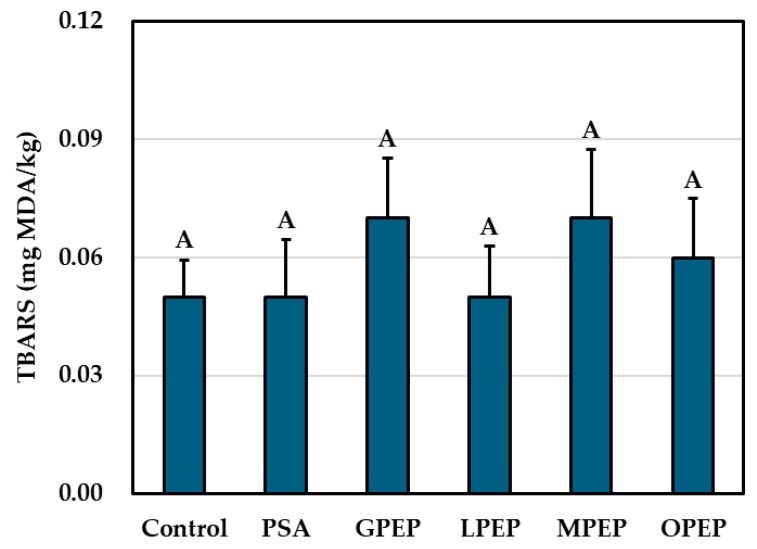
Effects of the citrus peel extract powders on TBARS of clean-label sausages: Control, 0.01% NaNO_2_ and 0.01% sodium ascorbate; PSA, 0.44% pre-converted Chinese cabbage powder and 0.01% sodium ascorbate; GPEP, 0.44% pre-converted Chinese cabbage powder and 0.1% grapefruit peel extract powder; LPEP, 0.44% pre-converted Chinese cabbage powder and 0.1% lemon peel extract powder; MPEP, 0.44% pre-converted Chinese cabbage powder and 0.1% mandarin peel extract powder; OPEP, 0.44% pre-converted Chinese cabbage powder and 0.1% orange peel extract powder. ^A^ Same superscript letter within a treatment denotes no significant differences (*p* > 0.05). The bar in each treatment indicates the standard error of the mean.

Lipid oxidation is a serious issue in processed meat products, affecting their flavor, color, and overall quality [54,55]. The TBARS assay measures malondialdehyde (MDA), a secondary oxidation product, and provides a reliable indication of the oxidative stability of meat [54,56]. No significant differences (*p* > 0.05) were observed in the TBARS values among the samples containing citrus peel extract powders, the nitrite-added control, and the PSA treatment (Figure 3), suggesting that both PCCP and the citrus peel extract powders contributed to oxidative stability. PCCP likely played a key role in inhibiting lipid oxidation by generating nitric oxide, thus providing a natural source of nitrite, which interacts with the lipid radicals and myoglobin and prevents oxidative degradation [40,57,58]. Vegetable-based nitrate sources have demonstrated similar antioxidant effects in naturally cured meat products, thereby reducing lipid oxidation [39,59,60]. Citrus peel extract powders provided additional antioxidant benefits, owing to their high polyphenol content (particularly flavonoids such as hesperidin and naringin), which scavenge free radicals and chelate pro-oxidant metal ions, thereby mitigating lipid oxidation [19,21,47,54]. Studies have shown that lemon and orange peel extracts effectively reduce the TBARS values and enhance the oxidative stability of meat products [17,27].

Our findings suggest that PCCP primarily contributes to oxidative stability through nitric oxide formation, while citrus peel extract powders provide supplementary antioxidant protection. This combination presents a promising clean-label alternative to synthetic additives, ensuring the oxidative stability of naturally cured meat products, while supporting consumer demand for natural ingredients.

## 4. Conclusions

In this study, we investigated the potential of citrus peel extract powder as an alternative reducing agent for naturally cured pork sausages formulated with PCCP. The incorporation of citrus extracts afforded a higher residual nitrite content than the control and sodium ascorbate-treated sausages, while maintaining comparable levels of cured meat pigment, total pigment, and curing efficiency. Although citrus peel extracts exhibited antioxidant properties, their effects on lipid oxidation were not significantly different among the treatments. Instrumental color analysis indicated that the citrus peel extracts increased yellowness without altering the redness or lightness of the sausages. Our findings suggest that citrus peel extract powders combined with PCCP can serve as natural curing accelerators without negatively affecting the development of the cured color or oxidative stability. However, further studies are required to assess their impact on microbial safety, sensory characteristics, and storage stability for commercial applications.

## Figures and Tables

**Table 1:** Analysis of pH, total phenolic content, and vitamin C content in citrus peel extract powders.

Citrus Peel Extract Powders ^1^	pH	Total Phenolic Content (mg GAE/g)	Vitamin C Content (mg AA/100 g)
GPEP	5.32 ± 0.01 ^B^	107.63 ± 0.48 ^A^	441.36 ± 11.33 ^C^
LPEP	4.90 ± 0.01 ^D^	58.71 ± 0.30 ^B^	874.84 ± 12.28 ^A^
MPEP	5.38 ± 0.01 ^A^	53.46 ± 0.26 ^C^	556.43 ± 16.09 ^B^
OPEP	5.11 ± 0.01 ^C^	53.29 ± 0.07 ^C^	455.55 ± 30.57 ^C^

All values are expressed as means ± standard error. ^A–D^ Different superscript letters within a column denote significant differences (*p* < 0.05). ^1^ Citrus peel extract powders: GPEP, grapefruit peel extract powder; LPEP, lemon peel extract powder; MPEP, mandarin peel extract powder; OPEP, orange peel extract powder.

**Table 2:** Effects of the citrus peel extract powders on cooking loss, pH, and CIE color of naturally cured pork sausages.

Treatments ^1^	Cooking Loss (%)	pH	CIE L*	CIE a*	CIE b*
Control	5.93 ± 0.18 ^A^	6.11 ± 0.01 ^A^	66.44 ± 0.14 ^A^	11.13 ± 0.08 ^A^	7.32 ± 0.03 ^C^
PSA	4.83 ± 0.09 ^CD^	6.12 ± 0.01 ^A^	65.99 ± 0.18 ^AB^	11.04 ± 0.07 ^A^	7.38 ± 0.03 ^C^
GPEP	4.98 ± 0.12 ^CD^	6.12 ± 0.01 ^A^	65.97 ± 0.16 ^AB^	11.00 ± 0.05 ^A^	7.64 ± 0.04 ^AB^
LPEP	4.54 ± 0.10 ^D^	6.11 ± 0.01 ^A^	65.98 ± 0.18 ^AB^	10.96 ± 0.07 ^A^	7.63 ± 0.03 ^AB^
MPEP	5.08 ± 0.15 ^BC^	6.11 ± 0.01 ^A^	65.83 ± 0.26 ^B^	10.95 ± 0.10 ^A^	7.66 ± 0.04 ^A^
OPEP	5.45 ± 0.19 ^B^	6.10 ± 0.02 ^A^	65.74 ± 0.19 ^B^	10.95 ± 0.06 ^A^	7.55 ± 0.02 ^B^

All values are expressed as means ± standard error. ^A–D^ Different superscript letters within a column denote significant differences (*p* < 0.05). ^1^ Treatments: Control, 0.01% NaNO_2_ and 0.01% sodium ascorbate; PSA, 0.44% pre-converted Chinese cabbage powder and 0.01% sodium ascorbate; GPEP, 0.44% pre-converted Chinese cabbage powder and 0.1% grapefruit peel extract powder; LPEP, 0.44% pre-converted Chinese cabbage powder and 0.1% lemon peel extract powder; MPEP, 0.44% pre-converted Chinese cabbage powder and 0.1% mandarin peel extract powder; OPEP, 0.44% pre-converted Chinese cabbage powder and 0.1% orange peel extract powder.

**Table 3:** Effects of the citrus peel extract powders on residual nitrite, cured meat pigment, total pigment, curing efficiency, and cured color intensity of naturally cured pork sausages.

Treatments ^1^	Residual Nitrite (ppm)	Cured Meat Pigment (ppm)	Total Pigment (ppm)	Curing Efficiency (%)	Cured Color Intensity (%R650/%R570)
Control	29.88 ± 4.91 ^B^	36.98 ± 0.99 ^A^	47.26 ± 0.13 ^A^	78.28 ± 2.31 ^A^	2.07 ± 0.02 ^A^
PSA	34.16 ± 5.68 ^B^	36.90 ± 0.87 ^A^	46.41 ± 0.11 ^AB^	79.53 ± 1.96 ^A^	2.05 ± 0.03 ^A^
GPEP	54.42 ± 0.26 ^A^	35.02 ± 0.80 ^A^	46.24 ± 0.26 ^B^	75.68 ± 1.30 ^A^	2.01 ± 0.04 ^A^
LPEP	54.17 ± 0.16 ^A^	34.95 ± 0.88 ^A^	46.41 ± 0.28 ^AB^	75.33 ± 2.02 ^A^	1.99 ± 0.04 ^A^
MPEP	53.91 ± 0.28 ^A^	34.73 ± 0.98 ^A^	46.75 ± 0.46 ^AB^	74.19 ± 1.38 ^A^	2.04 ± 0.03 ^A^
OPEP	54.98 ± 0.09 ^A^	34.15 ± 1.07 ^A^	46.24 ± 0.41 ^B^	73.75 ± 1.71 ^A^	2.03 ± 0.03 ^A^

All values are expressed as means ± standard error. ^A,B^ Different superscript letters within a column denote significant differences (*p* < 0.05). ^1^ Treatments: Control, 0.01% NaNO_2_ and 0.01% sodium ascorbate; PSA, 0.44% pre-converted Chinese cabbage powder and 0.01% sodium ascorbate; GPEP, 0.44% pre-converted Chinese cabbage powder and 0.1% grapefruit peel extract powder; LPEP, 0.44% pre-converted Chinese cabbage powder and 0.1% lemon peel extract powder; MPEP, 0.44% pre-converted Chinese cabbage powder and 0.1% mandarin peel extract powder; OPEP, 0.44% pre-converted Chinese cabbage powder and 0.1% orange peel extract powder.

## Data Availability

The original contributions presented in the study are included in the article, further inquiries can be directed to the corresponding author.

## Abbreviations

The following abbreviations are used in this manuscript:

PCCP	Pre-converted Chinese cabbage powder
GPEP	Grapefruit peel extract powder
LPEP	Lemon peel extract powder
MPEP	Mandarin peel extract powder
OPEP	Orange peel extract powder
GAE	Gallic acid equivalents
AA	Ascorbic acid
TBARS	Thiobarbituric acid reactive substances
MDA	Malondialdehyde
TPC	Total phenolic content
